# Soluble SORL1 in cerebrospinal fluid as a marker for functional impact of rare *SORL1* variants

**DOI:** 10.1002/alz.71042

**Published:** 2026-02-13

**Authors:** Matthijs W. J. de Waal, Sven J. van der Lee, Melanie Lunding, Lynn Boonkamp, Nolan Barrett, Giulia Monti, Anne Mette G. Jensen, Christian B. Vaegter, Jan Raska, Sona Cesnarikova, Jiri Sedmik, Calvin Trieu, Marjan M. Weiss, Rosalina van Spaendonk, Lisa Vermunt, Georgii Ozhegov, Niccolo Tesi, Marc Hulsman, Dovile Januliene, Arne Moller, Dasa Bohaciakova, Wiesje M. van der Flier, Olav M. Andersen, Charlotte E. Teunissen, Henne Holstege

**Affiliations:** ^1^ Genomics of Neurodegenerative Diseases and Aging, Human Genetics, Amsterdam UMC Vrije Universiteit Amsterdam Amsterdam The Netherlands; ^2^ Neurochemistry Laboratory, Department of Laboratory Medicine, Amsterdam UMC Vrije Universiteit Amsterdam Amsterdam The Netherlands; ^3^ Neurodegeneration Amsterdam Neuroscience Amsterdam The Netherlands; ^4^ Alzheimer Center Amsterdam, Neurology, Amsterdam UMC Vrije Universiteit Amsterdam Amsterdam The Netherlands; ^5^ Dept. of Biomedicine Aarhus University Aarhus Denmark; ^6^ Department of Histology and Embryology, Faculty of Medicine Masaryk University Brno Czech Republic; ^7^ International Clinical Research Center St. Anne's Faculty Hospital Brno Czech Republic; ^8^ Department of Human Genetics Radboud University Medical Centre Nijmegen The Netherlands; ^9^ Genome Analysis Laboratory Human Genetics, Amsterdam UMC location VUmc Amsterdam The Netherlands; ^10^ Delft Bioinformatics Lab Delft University of Technology Delft The Netherlands; ^11^ Department of Structural Biology University of Osnabrück, Neuer Graben/Schloss Osnabrück Germany; ^12^ Alzheimer Nederland Amersfoort The Netherlands; ^13^ Epidemiology and Data Science Vrije Universiteit Amsterdam, Amsterdam UMC location VUmc Amsterdam The Netherlands; ^14^ Amsterdam Public Health Amsterdam UMC Amsterdam The Netherlands; ^15^ VIB Center for Brain & Disease Research VIB Leuven Belgium; ^16^ Department of Neurosciences Leuven Brain Institute, KU Leuven Leuven Belgium

**Keywords:** Alzheimer's Disease, cerebrospinal fluid (CSF), missense variants, quantitative immunoassay, soluble SORL1 (sSORL1), *SORL1*

## Abstract

**INTRODUCTION:**

The sortilin‐related receptor (SORL1) directs APP and Aβ trafficking within the retromer pathway. Cleavage at the cell surface releases soluble SORL1 (sSORL1) into cerebrospinal fluid (CSF). We examined whether CSF‐sSORL1 can serve as an in vivo marker of genetically impaired SORL1.

**METHODS:**

CSF‐sSORL1 was quantified by enzyme‐linked immunosorbent assay (ELISA) in 218 participants: 90 carriers of *SORL1* variants, 78 *SORL1*‐wildtype (WT) AD patients, and 50 *SORL1*‐WT controls.

**RESULTS:**

sSORL1 concentrations were significantly lower in carriers of protein‐truncating and damaging missense variants. In *SORL1*‐WT patients, CSF‐s*SORL1* correlated with pTau181 but not with Aβ42 among AD patients, and did not differ between patients and controls.

**DISCUSSION:**

These findings suggest that impaired SORL1 trafficking reduces receptor delivery to the cell surface and thereby decreases sSORL1 shedding, supporting its potential use as a pathway‐specific biomarker.

**Highlights:**

- Enzyme‐linked immunosorbent assay (ELISA) enables quantitative measurement of soluble sortilin‐related receptor (sSORL1) in cerebrospinal fluid (CSF).
- sSORL1 levels are reduced in CSF from carriers of a pathogenic *SORL1* variant.
- CSF‐sSORL1 levels correlate with tau pathology in Alzheimer's disease.
- sSORL1 levels represent an in vivo biomarker of SORL1 function.

## 1 BACKGROUND

Sortilin‐related receptor (SORL1 also known as SORLA and LR11) operates in the endolysosomal pathway as a sorting receptor of various proteins, with a main function to traffic cargo proteins from the endosome to the Golgi or for recycling of cargo from the endosome back to the cell surface.^1^, ^2^ Genetic variants that impair SORL1 function have been linked with an increased risk of developing Alzheimer's disease (AD).^3^, ^4^, ^5^ In the context of AD, SORL1 can regulate the proteolysis of amyloid precursor protein (APP) into amyloid‐beta (Aβ) by controlling the transport of APP to the endosome.^6^, ^7^, ^8^, ^9^, ^10^ In addition, SORL1 facilitates the trafficking of cargo‐Aβ to lysosome for degradation.^11^ *SORL1* protein‐truncating variants (PTVs) have been observed almost exclusively in AD cases, supporting *SORL1* haploinsufficiency.^3^, ^12^ However, the majority of *SORL1* variants are rare missense variants, with diverse effects on SORL1 function.^13^, ^14^ While most variants are likely benign, a subset of variants have strong risk‐increasing effects on AD.^12^

Determining of the pathogenicity of *SORL1* variants is challenging, as variants are rare and pedigrees of *SORL1* variant carriers are commonly small.^15^, ^16^, ^17^, ^18^ We recently proposed a prioritization scheme of *SORL1* missense variants according to a “disease mutation domain mapping” approach (DMDM), a comprehensive manual effort, taking in‐depth knowledge of SORL1 functional domains into account.^12^ Using this approach, we leveraged mutations in proteins associated with monogenic diseases that share homologous domains with SORL1 to identify domain positions where mutations are predicted to be pathogenic. This led to the categorization of missense variants into high–moderate, low‐, and no‐priority missense variants. Testing the effect on AD risk in a large sequencing dataset from AD cases and controls ^12^ indicated that high‐priority variants associated with a 10‐fold increased risk of early onset AD and a 6‐fold increased risk of overall AD. In contrast, moderate‐, low‐, and no‐priority missense variants did not associate with AD risk. In addition to DMDM, variant prioritization may further benefit from a functional readout to support DMDM prediction of pathogenicity. Here, we leveraged the observation that functional SORL1 traffics to the cell‐surface, where the ectodomain of the receptor is cleaved by ADAM17 (TACE) into extracellular sSORL1, which is released in the interstitial space and cerebrospinal fluid (CSF).^19^ Impaired trafficking of the SORL1 receptor might be reflected in lower levels of SORL1 that reach the cell surface and, thus, lower levels of cleaved sSORL1 as recently shown using Western blotting (WB).^20^ Therefore, we explored whether ELISA for CSF sSORL1 can serve as an effective quantitative in vivo biomarker for pathogenic *SORL1* missense mutations. We first analytically validated a commercially available immunoassay to measure sSORL1 in CSF. Next, we measured sSORL1 concentrations in human CSF samples from AD patients who carried *SORL1* genetic variants with varying predicted pathogenicity, as well as from non‐variant carriers. Finally, we compared these CSF concentrations with those measured using WB to assess the consistency in our findings.

## 2 METHODS

### 2.1 Study cohort

**FIGURE 1 alz71042-fig-0001:**
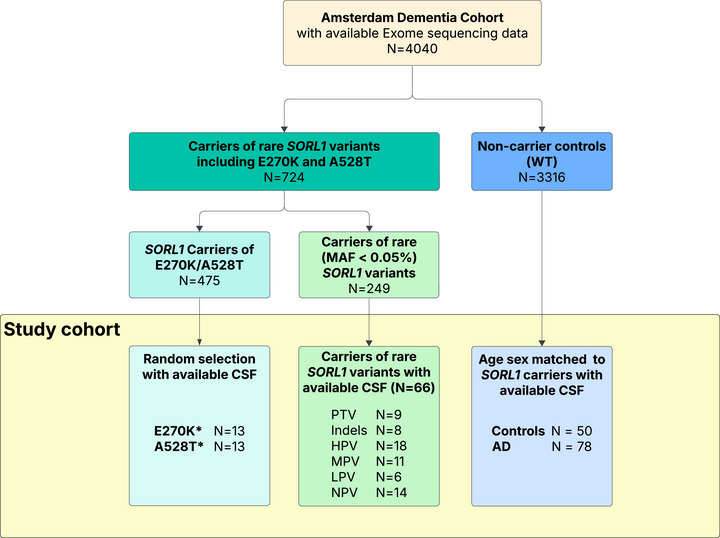
Selection of the study cohort. Participants were included from the Amsterdam Dementia Cohort. *SORL1* variant carrier status was determined using whole‐exome sequencing. Samples for which CSF was available were included in the study cohort, WT‐*SORL1* included controls, and AD patients (amyloid‐confirmed). A528T, p.Ala528Thr; CSF, cerebrospinal fluid; E270K, p.Glu270Lys; HPV, high priority variants; indels, in‐frame insertion/deletion; LPV, low priority variant; MAF, minor allele frequency; MPV, moderate priority variant; NPV, no priority variant; PTV, protein truncating variant; WT, wild‐type. *Two carriers both carried an E270K and A528T mutation and were included in both groups.

For this study, we selected 218 participants from the Amsterdam Dementia Cohort (ADC), which includes patients who visited the memory clinic at the Alzheimer Center, Amsterdam University Medical Center, The Netherlands, and underwent a diagnostic work‐up.^21^ We selected participants with available CSF samples and genetic information and divided them into three groups (see Figure 1). The first group consisted of 90 participants carrying rare genetic variants in the *SORL1* gene. Because our primary aim was to evaluate sSORL1 levels as a functional readout of *SORL1* variant effects, all variant carriers were included irrespective of diagnosis. Most participants within this group were diagnosed with dementia due to AD (*n* = 71), while others had diagnoses of mild cognitive impairment (MCI) (*n* = 9), vascular dementia (*n* = 2), psychiatric disorder (*n* = 2), subjective cognitive decline (SCD) (*n* = 5), and non‐neurodegenerative neurological disease (*n* = 1). Clinical diagnosis was made through consensus‐based, multidisciplinary meeting. AD‐type dementia diagnosis was according to the clinical criteria formulated by the National Institute of Neurological and Communicative Disorders and Stroke–Alzheimer's Disease and Related Disorders Association (NINCDS‐ADRDA) and based on National Institute of Aging–Alzheimer association (NIA‐AA).^22^, ^23^ AD diagnoses were supported by biomarker confirmation of amyloid‐positive status,^24^ determined through CSF analysis (*n* = 55) or positron emission tomography (PET) imaging (*n* = 15), except for one carrier whose amyloid status could not be confirmed. MCI diagnosis was based on international consensus criteria ^25^ and vascular dementia was diagnosed according to the NINDS–Association Internationale pour la Recherche et l'Enseignement en Neurosciences (NINDS‐AIREN) criteria.^26^ Diagnoses for psychiatric or non‐neurodegenerative neurological disorders were based on the diagnostic work‐up. The second group consisted of 78 *SORL1* wild‐type (WT) AD patients (65 CSF and 13 PET confirmed amyloid‐positive) that were selected to match optimally with the 90 carriers by age and sex. The third group consisted of 50 randomly selected cognitively normal *SORL1* WT controls, all of whom showed no signs of cognitive impairment during their diagnostic work‐up. Cognitive impairment was assessed using the Mini‐Mental State Examination (MMSE),^27^ with data available for all patients. In addition, apolipoprotein E (*APOE)* genotype information was available for all patients. All patients gave informed consent for the use of their medical data and biomaterial. The Medical Ethics Committee of the Amsterdam UMC approved this study in accordance with the declaration of Helsinki.

**RESEARCH IN CONTEXT**

1. **Systematic review**: Genetic studies have established strong associations between sortilin‐related receptor (*SORL1)* variants and Alzheimer's disease (AD) risk, but whether these variants lead to measurable functional effects in vivo remains unclear. Cellular studies suggest effects for a few variants, while previous biomarker work using Western blot provided only semi‐quantitative readouts and focused on limited variant types. A quantitative‐based approach across a broader range of variants has not yet been applied.
2. **Interpretation**: Using enzyme‐linked immunosorbent assay (ELISA) as a quantitative assay, we demonstrate that cerebrospinal fluid (CSF) concentrations of soluble SORL1 (sSORL1) are significantly reduced in carriers of pathogenic *SORL1* variants compared with wild‐type AD patients and controls. CSF‐sSORL1 also correlates with tau pathology, suggesting that SORL1‐retromer activity may be upregulated under AD‐related stress but impaired in variant carriers.
3. **Future directions**: Larger and independent cohorts should be used to replicate our findings and assess additional *SORL1* variants not covered here. Longitudinal studies are warranted to determine whether CSF‐sSORL1 fluctuations differ between carriers and non‐carriers over the course of disease.

### 2.2 *SORL1* genetic variant detection

*SORL1* variants were determined by whole exome sequencing (WES)^4^, ^5^ and stratified into different pathogenicity categories according to previously described selection criteria.^12^ Protein truncating variants (PTVs), included in‐frame insertion/deletion variants (in‐frame indels) and nonsense variants. Missense variants were stratified according to previously described DMDM,^12^, ^28^ into high priority missense variants (HPV), moderate priority missense variants (MPV), low priority missense variants (LPV), and no priority missense variants (NPV). Since no clear disease‐associated mutations have been found in any of the VPS10p family members, it was not possible to perform DMDM for the VPS10p/10CC domain, such that HPVs in this domain were based on having a REVEL score > 0.5 and MAF (> 0.05%; gnomAD).^29^, ^30^, [Fig alz71042-fig-0001]

### 2.3 CSF sample collection and measures

CSF samples were obtained as part of routine clinical care by lumbar puncture using a 20/25‐gauge needle and syringe between the L3/L4, L4/L5, or L5/S1 intervertebral space, collected in polypropylene tubes, and processed as previously described.^31^ Concentrations of the CSF AD core biomarkers (Aβ42 (*n* = 216), phosphorylated Tau‐181 (pTau‐181; *n* = 162), and total Tau (tTau; *N* = 162)) were measured in the Amsterdam UMC using commercial kits: Elecsys Aβ42, pTau (181P), and tTAU CSF assays (Roche Diagnostics, Basel, Switzerland) or INNOTEST β‐Amyloid(1‐42), pTau (181P), and hTAU Ag (Fujirebio, Gent, Belgium). Aβ42 concentrations measured on INNOTEST were corrected for a drifting effect in biomarker concentrations that appeared throughout the years.^32^ Abnormal amyloid status (A+) was determined based on previously defined abnormal assay‐specific concentration cutoffs in CSF: for INNOTEST, an Aβ42 level of <813 pg/mL was considered abnormal, while for Elecsys, an a pTau‐181/Aβ42 ratio of ≥0.02 was considered abnormal.^32^, ^33^

### 2.4 Validation and application of SORL1 ELISA in CSF

The quantification of sSORL1 was performed with a commercially available ELISA kit (ab282864, ABCAM, Cambridge, UK) according to the manufacturer's protocol, which was validated for Heparin Plasma, Cell Culture Supernatant, Serum, Cell Lysate, and ethylenediaminetetraacetic acid (EDTA) Plasma. Here, we analytically validated this assay for 10x diluted CSF, following standard protocols.^34^ We evaluated the assays’ precision, parallelism, dilution linearity, recovery, specificity, target sensitivity, and sample stability. Details of the different parameters can be found in the Supplementary Materials. This includes information on cell culture and SORL1 constructs used for specificity. Absorbance was measured with the EPOCH2NS reader (BioTek, Agilent, Santa Clara, US) using Gen5 reader software (Version 3.12). After validation, we applied the assay to 218 CSF samples as analyzed here.

### 2.5 WB analysis

For 37 *SORL1* variant carriers, we compared the ELISA results with the relative sSORL1 CSF concentrations (rel‐sSORL1) determined by WB. We determined the sSORL1 CSF concentrations in the variant carriers relative to concentrations of optimally matched with *SORL1* WT AD patients, based on gender, *APOE* genotype, and age at AD onset (Table S1). Details of the estimation of the rel‐sSORL1 concentration and generation of sSORL1 standard protein are described in the Supplementary Materials. In short, equal volumes of CSF or sSORL1 standard protein were separated by sodium dodecyl sulfate—polyacrylamide gel electrophoresis (SDS‐PAGE) using NuPAGE 4‐12% Bis Tris gels (NP0321BOX, Thermo Fischer, Waltham, US) and then transferred to nitrocellulose membranes (Amersham GE Healthcase Life Sciences, Chicago, US). Unspecific binding was blocked (0.25 M Tris‐Base, 2.5 M NaCl, 2% skimmed milk powder, 2% Tween‐20) for 1 h at room temperature, and incubated overnight at 4°C with mouse anti‐LR11 primary antibody (611860, BD Biosciences, Franklin Lakes, US). The following day, the membranes were incubated in polyclonal rabbit anti‐mouse horseradish peroxidase (HRP) secondary antibody (P0260, 1:1500; Agilent Dako) for 1 h at room temperature. After washing membranes in washing buffer (0.2 mM CaCl_2_, 0.1 mM MgCl_2_, 1 mM HEPES, 14 mM NaCl, 0.2% skimmed milk powder, 0.05% Tween 20) for a total of 25 min, proteins were detected with SuperSignal West Femto Maximum Sensitivity Substrate (34094, Thermo Fisher), and images were captured using LAS‐4000 chemiluminiscent imager (GE Healthcare). Signals were quantified with Multi Gauge software (Fujifilm Life Sciences, Tokyo, Japan).

**TABLE 1:** Demographics of the cohort.

Parameter	AD *SORL1* WT	Controls	PTV	High priority	In‐frame indels	Low priority	Moderate priority	No priority	p.A528T	p.E270K
N	78	50	9	18	8	6	11	14	13^a^ ^)^	13^a)^
Sex (F%)	52 (67%)	19 (38%)	4 (44%)	8 (44%)	3 (38%)	3 (50%)	6 (55%)	7 (50%)	8 (62%)	7 (54%)
Age (SD)	63.4 (7.1)	60.9 (6)	64.7 (8.3)	63.8 (6)	63 (5.5)	63.5 (8.8)	64.2 (7.1)	62.2 (7.3)	65.5 (6.2)	66.4 (8.3)
MMSE (SD)	20.7 (4.7)	28.3 (1.6)	23.4 (6.7)	20.7 (7.1)	22.8 (5.9)	21.5 (4.1)	22.9 (7.2)	20 (3.7)	19.8 (6.2)	21.8 (5)
Abeta‐42 (SD)	774 (266)	1511 (477)	695 (184)	848 (388)	934 (675)	650 (204)	1190 (482)	869 (365)	831 (298)	604 (176)
pTau (SD)	85.2 (32.3)	56.4 (18.1)	77.8 (16.2)	89.9 (32.8)	105.2 (33.1)	87.4 (26.5)	79.8 (40.1)	75.8 (50)	82.6 (37.8)	83 (22.6)
tTau (SD)	686 (354)	337 (115)	639 (236)	785 (402)	784 (393)	768 (257)	852 (866)	553 (354)	747 (419)	730 (266)
*APOE*‐4 (%)	37 (47%)	13 (26%)	5 (56%)	10 (56%)	5 (62%)	5 (83%)	7 (64%)	6 (43%)	8 (62%)	7 (54%)

Abbreviations: AD, Alzheimer's disease; APOE, apolipoprotein E; F, female; In‐frame indels, in‐frame insertion deletion variant; MMSE, Mini‐Mental State Examination; PTV, protein truncating variant; SD, standard deviation.

^a^ Two carriers have both an A528T variant and a E270K variant and are therefore included in both groups.

### 2.6 Statistical analysis

We analyzed the association between CSF‐sSORL1 concentrations and pathogenicity or *SORL1* variants in two ways. First, we used robust linear regression to investigate the relationship between CSF‐sSORL1 concentrations and variant‐effect on AD risk, as estimated for each of eight variant pathogenicity categories, while adjusting for age and sex. Rare missense variants were assigned to the (1) high‐, (2) moderate‐, (3) low‐, and (4) no‐ priority group, as estimated by DMDM in our previous study.^12^ Truncating variants were assigned to the (5) PTV group and individuals without *SORL1* variants were assigned to the (6) WT group. The risk level associated with each pathogenicity category was the natural logarithm of odds ratios associated with it, as estimated by our previous study.^12^

Second, we performed binary robust linear regression (adjusted for age and sex) to compare CSF‐sSORL1 concentrations across each pathogenicity category with those in the *SORL1* WT control group (*n* = 128), as well as with the AD WT group (*n* = 78) and cognitively unimpaired controls (*n* = 50). Statistical significance was set at a Bonferroni‐corrected threshold of 0.00625 (0.05/8 groups).

We performed causal mediation analysis to assess to what extent CSF sSORL1 levels mediate the association between *SORL1* variant carrier status (PTVs, HPVs) and AD risk. The mediator model regressed CSF sSORL1 on *SORL1* variant status, age, and sex, while the outcome model regressed AD status on these variables. Nonparametric bootstrap simulations (*n* = 2000) were used to estimate the mediation and direct effects.

Then we associated sSORL1 concentrations with (1) age (adjusted for sex), (2) sex (adjusted for age), (3) *APOE* genotypes, (4) MMSE, (5) AB42, (6) tTau, and (7) pTau‐181, using robust linear regressions adjusting for age and sex. The threshold for statistical significance was set at 0.00714, to correct for multiple testing (0.05/7 tests). In this analysis, we considered only *SORL1* WT AD cases, and excluded controls and *SORL1* mutation carriers, to minimize potential confounding effect from *SORL1*‐related deficiencies on AD pathology. Potential interactions of *APOE ε4* genotype on sSORL1 concentrations across carrier groups were calculated incorporating the interaction term between *APOE ε4* and the *SORL1* genotype groups (sSORL1 concentration ∼ pathogenicity category group * *APOE ε4* genotype).

We aimed to define the lower end of the normal range of sSORL1 concentrations. Values below this threshold should be enriched for pathogenic variant carriers. For this, we estimated the first percentile of sSORL1 concentrations (pg/mL) in the control group and its 95% confidence interval (CI). We performed a bootstrapping procedure with 1000 resampling iterations. In each iteration, we drew a new dataset by randomly sampling (with replacement) from the observed sSORL1 values in the control group, maintaining the original sample size. The first percentile was calculated for each bootstrap sample, and the 95% CI was determined as the 2.5th and 97.5th percentiles of the bootstrapped distribution. Relative sSORL1 concentrations in CSF quantified via WB, were compared against a reference median value of 100, representing the control group using Wilcoxon signed‐rank test.

## 3 RESULTS

### 3.1 SORL1 ELISA analytical validation

**FIGURE 2:**
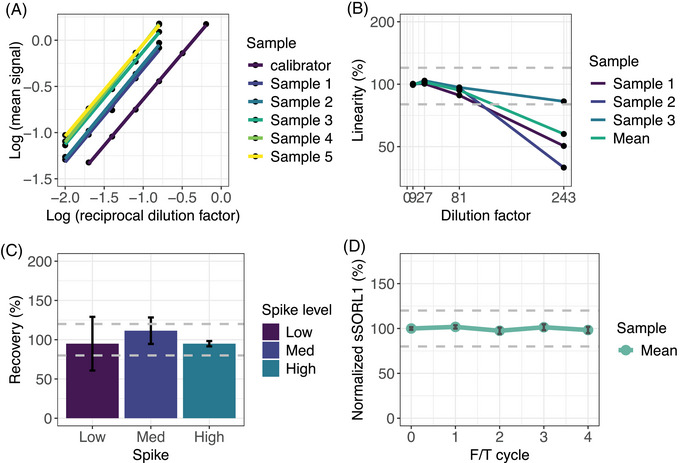
Technical validation of the CSF‐sSORL1 ELISA assay. (A) Parallelism indicates that two‐fold serial dilution of sSORL1 measurements are parallel to the measurement of the standard curve. (B) Dilution linearity indicates that samples remain reliably measurable within a range of 9‐fold to 81‐fold dilution. (C) The recovery of low, medium, and high spike concentrations falls within the expected range for accurate measurement. (D) Sample stability demonstrates that CSF samples subjected to four freeze/thaw cycles still yield similar concentrations. CSF, cerebrospinal fluid; ELISA, enzyme‐linked immunosorbent assay; sSORL1, soluble sortilin‐related receptor.

We tested the performance of the sSORL1 ELISA kit for CSF‐samples, a matrix that was not included in the specifications of the manufacturer. The lower limit of detection (LLOD) for this assay is 55 pg/mL. Precision of the assay in CSF was robust, with a 3.9% intra‐assay variability (low QC = 4.4%, medium QC = 3.4%, and high QC = 3.8 and a 10.0% inter‐assay variability (low QC = 12.0%, medium QC = 9.8%, and high QC = 8.2%). Parallelism (Figure 2A), linearity (Figure 2B), recovery (Figure 2C), and freeze‐thaw stability (Figure 2D) were all within the 85%–115% acceptable range. Thus, the assay allows the estimation of variation in CSF‐sSORL1 concentrations in CSF.

Then, we examined specificity/cross‐reactivity. According to the manufacturer, the antibodies for the ELISA assay were raised against a SORL1 recombinant fragment containing amino acid residue 82 to 755 of the SORL1 protein, which represents the VPS10p domain. This domain characterizes the VPS10p family of proteins (SORL1, SORT1, SORCS1, SORCS2, and SORCS3). To test for cross‐reactivity of the ELISA with other VPS10p family members, we spiked solutions of recombinant SORL1 protein with defined concentrations of recombinant SORT1 or SORCS2 (see Supplementary Materials). We did not observe an increase in the sSORL1 concentration after spiking (Figure S1), suggesting that there was no cross‐reactivity. In addition, the assay was able to detect different full size SORL1 recombinant proteins, including one produced in‐house (residue 82‐2135) and another commercially available (residue 82‐2135) (Figure S2); see details in Supplementary Materials. Finally, to determine potential nonspecific signal, we measured the media of different *SORL1* KO cell lines, which should be devoid of any sSORL1 protein. Indeed, we observed almost no signal for the KO condition, while the WT and the mock cell lines showed good expression (Figure S3). Overall, these results demonstrate the robustness and specificity of the assay for detecting sSORL1, without cross‐reactivity to soluble cleaved products of other VPS10p domain‐containing proteins.

### 3.2 High‐risk *SORL1* missense variants exhibit lower CSF‐sSORL1 levels similar to PTVs

To assess whether *SORL1* variant carriers had different sSORL1 concentrations compared to WT‐*SORL1* carriers, we measured sSORL1 concentrations in CSF derived from (1) 50 non‐demented controls (average age at sample collection is 61 (SD = 6), 38% female); (2) 78 *SORL1*‐WT AD patients (average age at sample collection is 63, 66% female); (3) 90 *SORL1* variant carriers (average age at sample collection is 64 (SD = 7), 50% female). See Table 1 for demographics.

We categorized the CSF samples from the 90 *SORL1* variant carriers according to predicted variant effects (listed in Table S1): 9 PTVs, 18 rare HPVs, 11 rare MPVs, 6 rare LPVs, rare 14 NPVs, rare 8 in‐frame indels, 11 carriers of the common E270K variant (rs117260922), 11 carriers of the common A528T variant (rs2298813), and 2 individuals who carried both the E270K and A528T common variants. The remaining 128 CSF samples were derived from individuals who were WT for *SORL1*. CSF sSORL1 concentrations varied widely among individuals (Figure S4), and were similar between *SORL1* WT‐AD (*n* = 78; mean: 462 ± 134 pg/mL) and *SORL1* WT‐NC (*n* = 50; mean: 474 ± 131 pg/mL) (*p* = 0.45) (Table S2), such that these groups were merged into a single “*SORL1* WT” control group for further analysis. Table S2 reports comparisons of the variant carrier groups with both the individual AD and control *SORL1* WT subgroups, as well as with the combined *SORL1* WT reference group used in the main analyses. Additionally, due to limited statistical power, carriers of two or three NPVs or two MPVs were grouped according to the highest predicted effect for further analysis.

We previously established that *SORL1* variants adhering to different priority groups associated with specific effects on AD risk (i.e. odds ratios [ORs]): OR_PTV_ = 17.2, OR_HPV_ = 6.1, OR_MPV_ = 1.5, OR_LPV_ = 1.2, OR_NPV_ = 1.1. This then allowed us to investigate variant‐associated sSORL1 concentrations relative to category‐specific AD‐risk. We found that carriers of variants with higher predicted risk for AD, have lower sSORL1 concentrations: for each 1‐unit increase in the natural log‐transformed odds ratio (ln(OR)), sSORL1 concentrations decreased by 87.4 pg/mL (*β* = ‐87.4; SE = 12.4; *p* = 1.9×10^−12^)(Figure S5). We performed causal mediation analysis to test to what extent the effect of pathogenic *SORL1* variants on AD risk is mediated through CSF sSORL1 levels. For PTVs and HPVs, the average causal mediation effect (ACME) was not statistically significant (PTVs: ACME = 0.044, *p* = 0.50; HPVs: ACME = 0.029, *p* = 0.50), whereas the average direct effect (ADE) remained significant (PTVs: ADE = 0.304, *p* = 0.001; HPVs: ADE = 0.280, *p* = 0.004), suggesting that the association between variant carriership and AD risk is largely independent of CSF sSORL1 concentrations.

**FIGURE 3:**
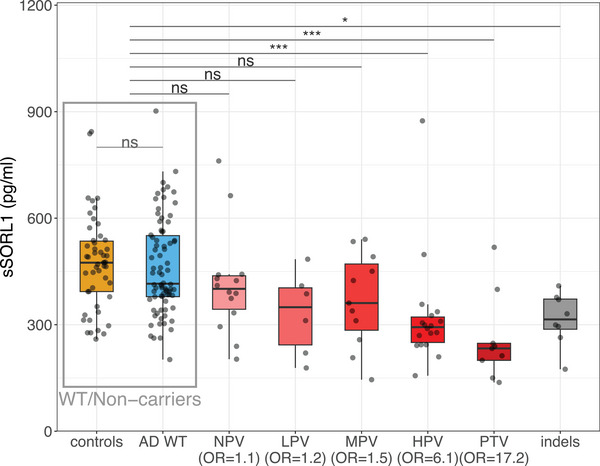
sSORL1 concentrations versus pathogenicity category. ELISA measurements of sSORL1 concentrations in CSF derived from carriers of *SORL1* genetic variants with different pathogenicity categories or carriers of WT *SORL1*. Sample size per group: Controls = 50, AD WT = 78, NPV = 14, LPV = 6, MPV = 11, HPV = 18, and PTV = 9. *p*‐Values were obtained using robust linear models adjusted for age and sex. CSF, cerebrospinal fluid; ELISA, enzyme‐linked immunosorbent assay; HPV, high priority variants; indels, in‐frame insertion deletion variant; LPV, low priority variant; MPV, moderate priority variant; NPV, no priority variant; OR, odds ratio; PTV, protein truncating variant; sSORL1, soluble sortilin‐related receptor; WT, wild‐type. Adjusted significance threshold: 0.000625 < *p*≤0.00625 = *; 0.0000625 < *p*≤0.000625 = **; *p*≤0.0000625 = ***.

**FIGURE 4:**
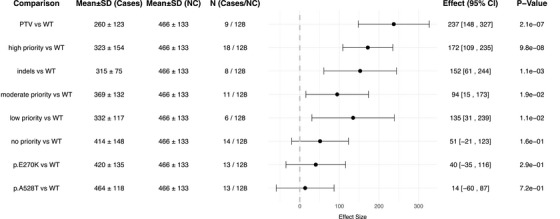
The effect of different severity groups versus WT. The effect having a specific *SORL1* variant on the CSF‐sSORL1 concentration, as determined by a robust linear regression for CSF‐sSORL1 concentrations between the different severity groups versus WT (*SORL1* WT AD cases + controls combined). AD, Alzheimer's disease; CI, confidence interval; CSF, cerebrospinal fluid; SD, standard deviation; SORL1, sortilin‐related receptor; sSORL1, soluble sortilin‐related receptor; WT, wild‐type.

Next, we compared CSF‐sSORL1 concentrations between individual variant groups and *SORL1* WT controls (Figures 3 and 4). The average CSF‐sSORL1 concentration across the 128 WT‐*SORL1* carriers was 466 pg/mL (SD = 133). As expected PTV carriers, who carry only one functional allele had significantly lower CSF‐sSORL1 concentrations compared to the group of *SORL1* WT carriers (i.e the merged *SORL1* WT AD cases and controls) (260 pg/mL ± 123 SD, *p* = 2.1×10^−^⁷). Notably, CSF‐sSORL1 concentrations of high‐priority missense variants were lowest of all prioritized missense variant groups, and significantly lower than the *SORL1* WT controls (323 pg/mL ± 154 SD, *p* = 9.8×10^−^ ^8^). Specifically, recent functional studies on the Y1816C and D1105H variants have demonstrated their pathogenicity and causative effect on AD ^17^, ^35^ and in the CSF of the carriers of these variants, we observed very low sSORL1 concentrations (305 pg/mL for Y1816C; 270 and 309 pg/mL for D1105H). In contrast, the sSORL1 concentrations of MPV, LPV, and NPV carriers were not significantly different from the WT controls (*p*_adj_ > 0.00625). In‐frame indels, for which AD risk estimates are currently unavailable and which cannot be assigned to any prioritization group, showed significantly reduced concentrations compared to WT controls (315 ± 75 pg/mL, *p* = 1.1×10^−3^). Finally, CSF‐sSORL1 concentrations in carriers of the E270K and A528T common variants were not significantly different compared to WT controls (E270K: 420 pg/mL ± 135 SD, *p* = 0.29; A528T: 464 pg/mL ± 118, *p* = 0.72).

The distribution of sSORL1 concentrations was wide, prompting us to estimate the lower bound (lowest 1%) of the CSF‐sSORL1 concentration range in WT *SORL1* controls. We found the lower‐bound CSF concentrations to be at 260 pg/mL (95% CI: 202‐274 pg/mL) sSORL1 concentrations below this lower‐bound extreme can be considered abnormally low. Notably, 12 out of 27 (44%) HPV and PTV carriers had sSORL1 levels below this threshold. In addition, several carriers of variants within the non‐pathogenic WT, MPV, LPV, and NPV categories had very low sSORL1 levels. Specifically, while variants translating to G1440R and S1107N, affecting the CR domain, were conservatively classified as a “moderate priority variant” due to a lack of evidence for pathogenicity by DMDM analysis,^12^ our results suggest that these variants may have an impairing effect on SORL1 function. On the other hand, we observed that several HPV and PTV carriers had unexpectedly high sSORL1 levels. For example, we observed an outlier high sSORL1 concentration in the CSF derived from a carrier of an exon 13‐splicing variant that likely causes an exon skipping frameshift, expected to lead to nonsense mediated RNA decay. Possibly, the incorrect splicing may be incomplete such that the haploinsufficiency observed for PTVs is less pronounced. Alternatively, in addition to impairing genetic variation, sSORL1 levels may be driven by alternative, currently unknown mechanisms.

### 3.3 Measurement of sSORL1 in CSF and cell culture media using WB and sSORL1 in plasma

We validated ELISA‐based sSORL1 measurements in CSF using WB in a subset of 37 individuals, observing a strong correlation (*ρ* = 0.54; *p* = 6.6 × 10^−^⁶; Figure S6A) given the distinct analytical methods underlying the two assays. When ELISA values were expressed as relative ratios (analogous to the WB approach), the correlation with WB‐derived measures increased (*ρ* = 0.64, *p* = 3.1 × 10^−^⁸; Figure S6B). WB images and comparisons across pathogenicity categories are provided in (Figures S7 and S8; uncropped blots of S7 are included as Supplementary Files).

In agreement with ELISA and WB, a cellular model expressing either high‐priority missense variants or in‐frame indels exhibited significantly reduced sSORL1 secretion compared to WT, also these models show no difference in sSORL1 secretion in media between cells expressing the common E270K and A528T variants and those expressing WT SORL1 (Supplementary Materials and Figure S9; uncropped blots of S9 are included as Supplementary File). Finally, for 44 individuals, both CSF and plasma samples were available, which indicated that plasma sSORL1 concentrations did not correlate with CSF‐sSORL1 concentrations (*ρ* = 0.126; Figure S10). A comparison between pathogenicity categories, is provided in Figure S11.

### 3.4 Association of CSF sSORL1 concentrations with *APOE* genotype, MMSE, age of diagnosis, p‐Tau181, t‐Tau, and A‐beta 42

**FIGURE 5:**
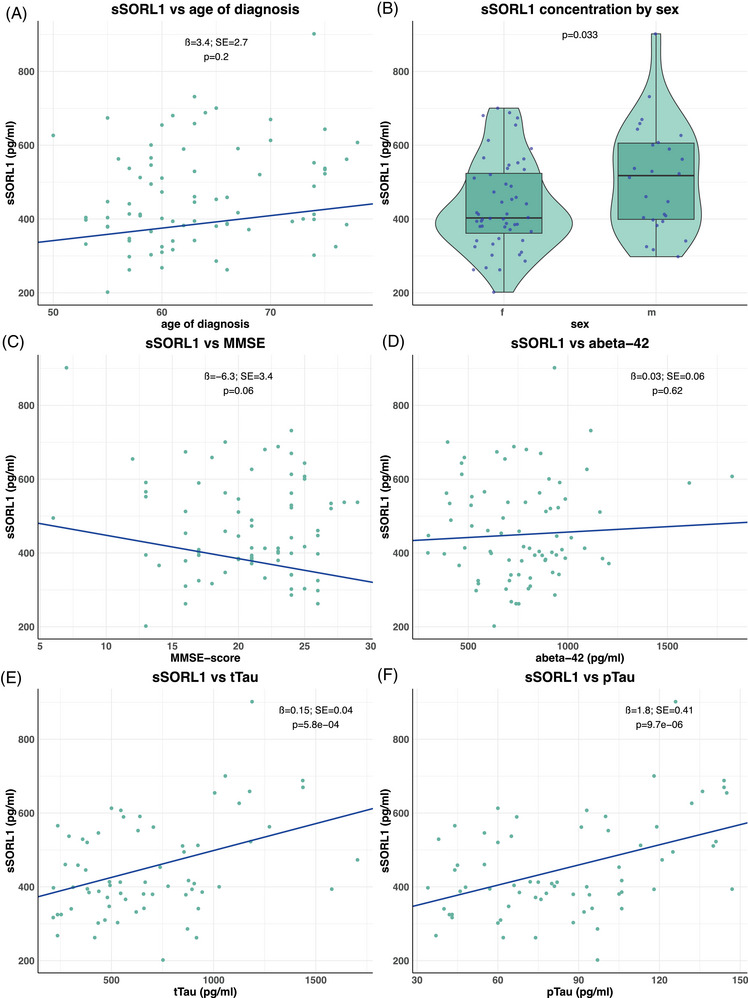
Correlation of s*SORL1* levels in *SORL1* WT AD patients (*n* = 78), with age of diagnosis, sex, MMSE, and AD biomarkers. To assess the relationship between CSF‐sSORL1 concentrations and age at diagnosis (A), sex (B). MMSE (C) and AD biomarkers (D)–(F) in *SORL1* WT AD, we performed a Robust Linear Model to account for the potential influence of outliers. The association of age of diagnosis with CSF‐sSORL1 was corrected for sex and *APOE*‐status. The association of sex with CSF‐sSORL1 was corrected for age of diagnosis. The association of MMSE and the AD biomarkers with CSF‐sSORL1 were corrected for age of diagnosis and sex. AD, Alzheimer's disease; CSF, cerebrospinal fluid; MMSE, Mini‐Mental State Examination; SORL1, sortilin‐related receptor sSORL1, soluble sortilin‐related receptor; WT, wild‐type.

Lastly, we investigated whether CSF‐SORL1 levels in *SORL1* WT AD patients were significantly associated with clinical measures of dementia and other CSF‐biomarker levels, including *APOE*‐ε4 genotype, MMSE, age, sex, Aβ42, tTau, and pTau‐181, accepting associations with *p*_adj_ < 0.00714 as significant. CSF‐sSORL1 levels were not significantly associated with age at AD diagnosis (*β* = 3.4; SE = 2.7; *p* = 0.21) (Figure 5A). Furthermore, we observed that females trended to have lower CSF‐sSORL1 levels (mean = 437 pg/mL) compared to males (mean = 511 pg/mL), although with a *p*‐value of 0.033 this was not accepted as a significant correlation (Figure 5B). Similarly, higher CSF‐sSORL1 levels trended to be associated with lower cognitive performance as measured by MMSE score, but this did not reach significance (*β* = −6.3; SE = 3.4; *p* = 0.06) (Figure 5C). CSF‐sSORL1 did not associate with *APOE ε4* genotype (*β* = −7.6; SE = 20.6; *p* = 0.71; data not shown), and we observed no significant interaction effect between *APOE ε4* status and *SORL1* pathogenicity category (*p* = 0.19). While CSF‐sSORL1 did not associate with CSF‐Aβ42 concentrations (*β* = 0.03; SE = 0.06; *p* = 0.62) (Figure 5D) we observed a positive correlation with both tTau (*β* = 0.15; SE = 0.04; *p* = 5.8×10^−^⁴) and pTau‐181 (*β* = 1.8; SE = 0.41; *p* = 9.7×10^−^⁶), which seems to be driven by the subset of AD patients with the highest levels of CSF‐(p) and (t)Tau (Figure 5E,F). Then, we investigated to what extent correlations between CSF‐sSORL1 concentrations and AD biomarkers covered the wider cognitive spectrum. Upon correlating CSF‐sSORL1 across the union of AD cases and controls who were *SORL1* WT, we observed only a weak trend for an (unexpected) positive association with CSF‐Aβ42 concentrations (*p* = 0.022), and while the correlation with pTau‐181 was weaker than when conditioning on AD patients only, it remained significant (*p* = 3.1 × 10^−3^) (Figure S12).

## 4 DISCUSSION

CSF‐sSORL1 concentrations were strongly variable, and distributions overlapped between AD patients and healthy controls. However, CSF‐sSORL1 concentrations in carriers of high‐priority *SORL1* missense variants (HPVs) were significantly lower than those observed in *SORL1* WT AD patients or controls, and resembled concentrations observed in carriers of PTVs. These findings identify CSF‐sSORL1 as a potential biomarker that may aid in the interpretation of *SORL1* variant functional impact.

Specifically, CSF‐sSORL1 concentrations in HPV carriers clustered at the lower end of the distribution. Notably, carriers of the mechanistically validated pathogenic variants Y1816C and D1105H showed very low CSF‐sSORL1 levels,^17^, ^35^, ^36^ consistent with findings by Castelot et al., who demonstrated decreased CSF‐sSORL1 in carriers of “trafficking‐defective” variants using WB.^20^ Similarly, PTV carriers showed reduced CSF‐sSORL1 concentrations, which is consistent with expectations for losing one copy. Although PTVs and HPVs reduce CSF‐sSORL1, mediation analysis suggests this does not explain the increased risk of AD, suggesting that the more direct impairing effects of these variants on intracellular trafficking mechanisms are central to the predisposition to disease. Carriers of in‐frame indels also displayed significantly reduced sSORL1 concentrations than WT‐*SORL1* carriers, although these were slightly higher than PTV‐ and HPV‐carriers. This suggest that in‐frame indels may have a milder but still deleterious effect compared to PTV and HPV carriers. Several MPV carriers had very low CSF‐sSORL1 concentrations, suggesting that although variants were not predicted to be pathogenic by DMDM, they mays still have an impairing effect on SORL1 function. On the other hand, for some HPV and PTV we observe relatively high CSF‐sSORL1 levels. This variability suggests that additional factors, other than rare coding genetic variants, such as non‐coding and epigenetic differences between *SORL1* haplotypes^37^ or effects of sex and disease severity, may influence sSORL1 concentrations in CSF.

Although most individuals carrying *SORL1* variants were diagnosed with AD or prodromal conditions such as MCI or SCD, a few had other neurological or psychiatric diagnoses. These were included since our aim was to assess sSORL1 levels in *SORL1* variant carriers as a functional measure of variant effect, independent of clinical diagnosis. Given their small number, any influence on the overall results is likely minor, though some residual confounding related to disease type, disease stage, amyloid/tau pathology, vascular comorbidity, or medication use cannot be fully excluded and should be addressed in future studies.

Previous studies examining CSF‐sSORL1 in AD have reported inconsistent results. One ELISA‐based study found elevated sSORL1 levels in AD,^38^ while two WB studies showed conflicting outcomes: one reported decreased sSORL1 in patients with mild cognitive decline,^39^ and another observed no difference between AD and controls but reduced levels among carriers of pathogenic *SORL1* variants.^20^ Differences in assay methodology, cohort size, and disease stage (e.g., severity of cognitive decline) likely contribute to these discrepancies. In our study, we did not observe an AD‐related difference in CSF‐sSORL1 concentrations, consistent with the findings of Castelot et al.^20^ Our study extends these observations by employing a quantitative ELISA assay, enabling comparison of absolute concentrations across individuals, and by analyzing a broader range of *SORL1* variants classified using a gene‐wide prioritization scheme. Together, our findings further strengthen the evidence that *SORL1* variant pathogenicity, rather than AD diagnosis, underlies reduced CSF‐sSORL1 levels.

Upon investigation of associations between CSF‐sSORL1 levels and aspects of AD, we found that CSF‐sSORL1 concentrations did not correlate with CSF‐Aβ42 concentrations *within* our sample of amyloid positive *SORL1* WT AD patients, despite SORL1’s role in APP processing and Aβ degradation.^6^, ^7^, ^8^, ^9^, ^10^, ^11^ Thus, while CSF‐Aβ42 clearly distinguishes between AD cases and controls,^24^ CSF‐Aβ42 concentrations may not contribute additional meaningful signal within advanced (amyloid positive) AD cases,^40^ which might preclude any association with CSF‐sSORL1. Supporting this interpretation, a study in *SORL1*‐deficient minipigs that genetically model PTV carriers have shown the expected inverse relationship between CSF‐sSORL1 and Aβ levels before amyloid plaque formation,^41^ suggesting that this link may be masked in later disease stages. An alternative explanation may relate to distinct biological processes. sSORL1 shedding primarily reflects intracellular receptor trafficking and endolysosomal processing, whereas CSF‐Aβ42 levels mainly capture extracellular amyloid deposition. Consequently, these two markers may represent distinct molecular events within the amyloid pathway, and their relationship may weaken once extracellular amyloid pathology is established. In contrast, we observed a positive association between CSF‐sSORL1 with (p)Tau concentrations within the *SORL1*‐WT AD cases. Since elevated CSF (p)Tau levels are indicative of increased disease severity,^24^, ^42^ we speculate that higher sSORL1 concentrations reflect an adaptive cellular response to counter disease‐associated neurodegenerative stress, possibly linked to enhanced retromer activity. Recent in vitro work has suggested a possible interaction between SORL1 and tau that could influence tau seeding, propagation or aggregation.^43^ However, evidence for this mechanism remains limited, and the observed association should therefore be regarded as hypothesis‐generating and explored further in future studies. While the cross‐sectional design of our study precluded us from assessing fluctuations in CSF‐sSORL1 concentrations with different disease stages, this should clearly be an important objective for future studies.

Here, we provide the first thorough technical validation of an ELISA assay for sSORL1 in CSF. The ELISA detects the SORL1 VPS10p domain. No nonspecific signals were detected in *SORL1* knockout (KO) cells, and the assay showed no cross‐reactivity with other VPS10p family members (SORT1 and SORCS2). However, since the epitopes of the antibodies are not fully characterized, we cannot exclude that binding may depend on a properly folded VPS10p domain. While calibrated with a recombinant SORL1 VPS10p fragment, the assay successfully detected full‐length sSORL1. Importantly, CSF‐sSORL1 concentrations were consistent between ELISA and WB analyses across genetic variant carriers, reinforcing the robustness of sSORL1 detection in CSF. However, future studies should confirm the robustness and generalizability of the ELISA assay in independent datasets and across centers.

The investigation of CSF‐SORL1 concentrations in multiple individuals who carry the same rare mutation contributes to an increased understanding of robustness of specific variant‐effects on in CSF‐sSORL1,^17^, ^35^ but the extreme rarity of impairing *SORL1* variants precludes such an analyses for most. Here, we presented five carriers of the p.F1123‐R1124delinsLS variant (four AD patients and one SCD patient), in whom we observed consistent CSF‐sSORL1 concentrations, suggesting a robust variant‐specific effect on CSF‐sSORL1 levels. Furthermore, we found that variants, grouped according to having a similar predicted effect on AD risk, have a similar effect on CSF‐sSORL1 concentrations, further supporting the observation that genetic *SORL1* impairment may have a robust variant‐specific effect on CSF‐sSORL1 concentrations.

In conclusion, our findings suggest that CSF‐sSORL1, as quantified by the sSORL1 ELISA assay, may represent a biomarker for the assessment of the functional impact of specific rare *SORL1* variants, rather than a diagnostic biomarker for AD. Despite strong inter‐individual variability in CSF‐sSORL1 concentrations, our results show a robust decrease in CSF‐sSORL1 concentrations in carriers of rare impairing *SORL1* missense variants, comparable to the decrease observed in carriers of PTVs. Further refinement of the clinical utility of CSF‐sSORL1 in confirming variant pathogenicity is warranted, including further investigations on the impact of factors including disease status and sex on the wide variance in concentrations. Together, we hypothesize that genetic impairment of SORL1 leads to increased risk of AD due to the inability of the retromer to traffic cargo and to rescue early AD‐associated cell stress, predisposing variant carriers to the development of advanced AD processes. Whether on the other hand, enhanced *SORL1* expression may contribute to increased tau seeding and advancement of AD‐associated processes will have to be focus of future studies in larger cohorts with longitudinal design.

## AUTHOR CONTRIBUTIONS

*Conceived the study*: Olav M. Andersen, Charlotte E. Teunissen, Henne Holstege; *Wrote the manuscript*: Matthijs W. J. de Waal, Sven J. van der Lee, Jan Raska, Lisa Vermunt, Melanie Lunding, Dasa Bohaciakova, Wiesje M. van der Flier, Olav M. Andersen, Charlotte E. Teunissen, Henne Holstege; *Data acquisition*: Matthijs W. J. de Waal, Sven J. van der Lee, Melanie Lunding, Lynn Boonkamp, Nolan Barrett, Giulia Monti, Anne Mette G. Jensen, Christian B. Vaegter, Sona Cesnarikova, Jiri Sedmik, Calvin Trieu, Marjan M. Weiss, Rosalina van Spaendonk, Georgii Ozhegov, Dovile Januliene, Arne Moller, Dasa Bohaciakova; *Analyses*: Matthijs W. J. de Waal, Sven J. van der Lee, Melanie Lunding, Lynn Boonkamp, Nolan Barrett, Giulia Monti, Lisa Vermunt, Niccolo Tesi, Marc Hulsman, Wiesje M. van der Flier, Olav M. Andersen, Charlotte E. Teunissen, Henne Holstege. All authors read, revised, and approved the final manuscript.

## CONFLICT OF INTEREST STATEMENT

As of 1‐11‐2025, W.F. is Executive Director at Alzheimer Nederland, Amersfoort, the Netherlands. Before 1‐11‐2025, W.F. has been an invited speaker at Biogen MA Inc, Danone, Eisai, WebMD Neurology (Medscape), NovoNordisk, Springer Healthcare, European Brain Council. All funding is paid to her institution. W.F. has been consultant to Oxford Health Policy Forum CIC, Roche, Biogen MA Inc, Eisai, Eli Lilly, Owkin France, and Nationale Nederlanden Ventures. W.F. participated in advisory boards of Biogen MA Inc, Roche, and Eli Lilly. W.F. was member of the steering committee of phase 3 EVOKE/EVOKE+ studies (NovoNordisk, 2024‐2025) and, in 2025, of the phase 3 Trontinemab study (Roche). All funding was paid to Amsterdam UMC. W.F. is chair of the Scientific Leadership Group of InRAD. W.F. was associate editor of Alzheimer, Research & Therapy in 2020/2021 and associate editor at Brain from 2021–2025. W.F. is member of Supervisory Board (Raad van Toezicht) Trimbos Instituut. C.E.T. has research contracts with Acumen, ADx Neurosciences, AC‐Immune, Alamar, Aribio, Axon Neurosciences, Beckman‐Coulter, BioConnect, Bioorchestra, Brainstorm Therapeutics, C2N diagnostics, Celgene, Cognition Therapeutics, EIP Pharma, Eisai, Eli Lilly, Fujirebio, Instant Nano Biosensors, Merck, Muna, Novo Nordisk, Olink, PeopleBio, Quanterix, Roche, Toyama, Vaccinex, Vivoryon. She is editor in chief of Alzheimer Research and Therapy, and serves on editorial boards of Molecular Neurodegeneration, Alzheimer's & Dementia, Neurology: Neuroimmunology & Neuroinflammation, Medidact Neurologie/Springer, and is committee member to define guidelines for Cognitive disturbances, and one for acute Neurology in the Netherlands. She has consultancy/speaker contracts for Aribio, Biogen, Beckman‐Coulter, Cognition Therapeutics, Eisai, Eli Lilly, Merck, Novo Nordisk, Novartis, Olink, Roche, Sanofi, and Veravas. H.H. received consultancy fees from Retromer Therapeutics and Muna Therapeutics; all funding is paid to her institution. M.W.J.d.W., S.v.d.L., M.L., L.B., N.B., G.M., A.M.G.J., J.R., S.C., C.B.V., J.S., C.T., M.M.W., R.v.S., L.V., G.O., N.T., M.H., D.J., A.M., D.B., and O.M.A. have nothing to disclose. Author disclosures are available in the Supporting Information.

## CONSENT STATEMENT

All patients gave informed consent for the use of their medical data and biomaterial. The study was approved by the Medical Ethics Committee of Amsterdam UMC and conducted in accordance with the Declaration of Helsinki.

## Supporting information



Supporting information

Supporting information

Supporting information

Supporting information

## Data Availability

The data supporting the findings of this study are not publicly available due to sensitivity concerns. However, they can be accessed upon reasonable request from the corresponding author, subject to approval by the Medical Ethics Committee of Amsterdam UMC.
