# Supplementary material for: Soluble SORL1 in cerebrospinal fluid as a marker for functional impact of rare SORL1 variants
Source: Alzheimers Dement. 2026 Feb 13;22(2):e71042. doi: 10.1002/alz.71042 (PMC12902900; doi:10.1002/alz.71042)
Supplement: Supplementary file 3 — Supporting information [file ALZ-22-e71042-s004.pptx]

## Slide 1
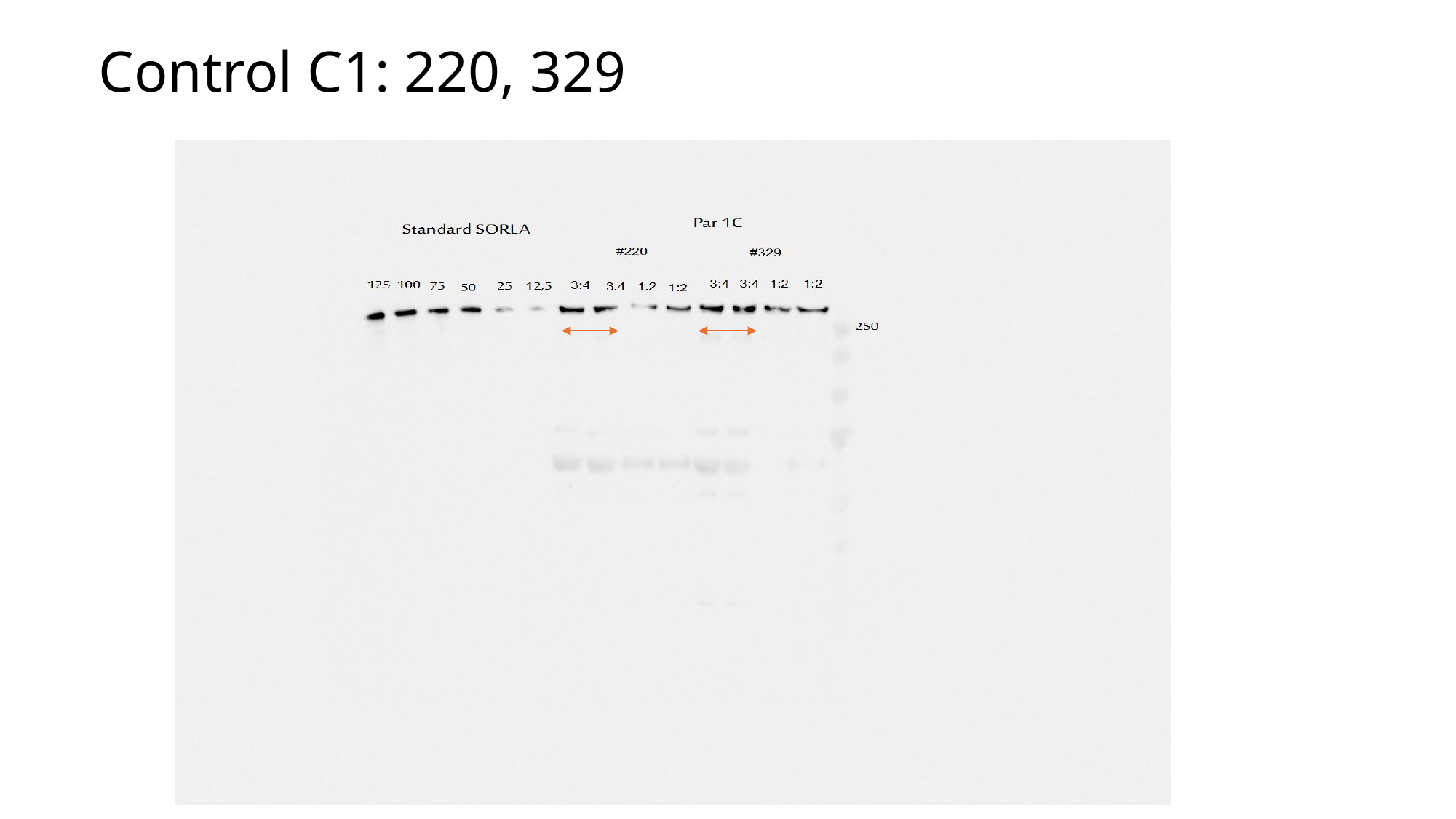

# Control C1: 220, 329

## Slide 2
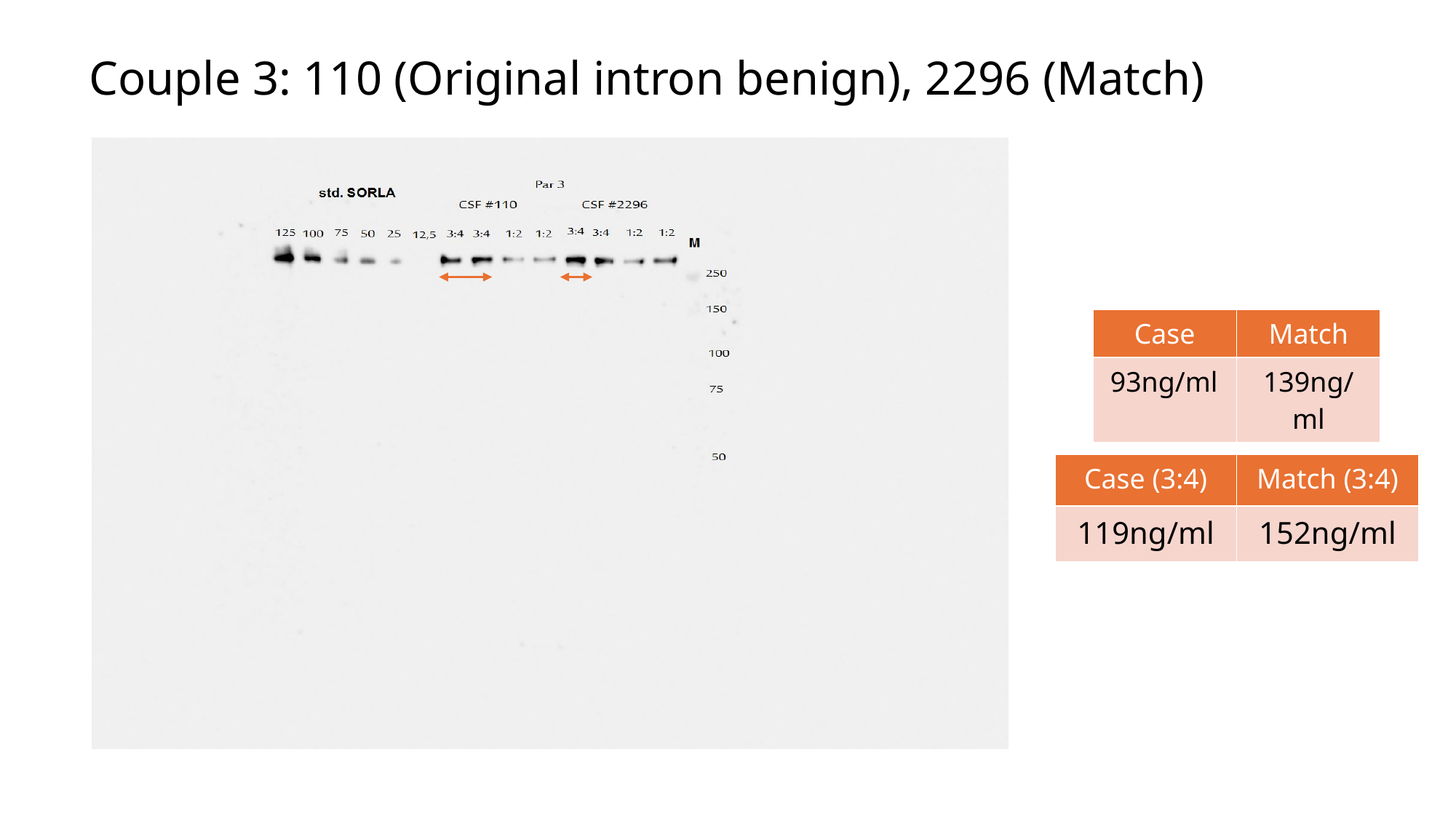

# Couple 3: 110 (Original intron benign), 2296 (Match)
| Case | Match |
| --- | --- |
| 93ng/ml | 139ng/ml |
| Case (3:4) | Match (3:4) |
| --- | --- |
| 119ng/ml | 152ng/ml |

## Slide 3
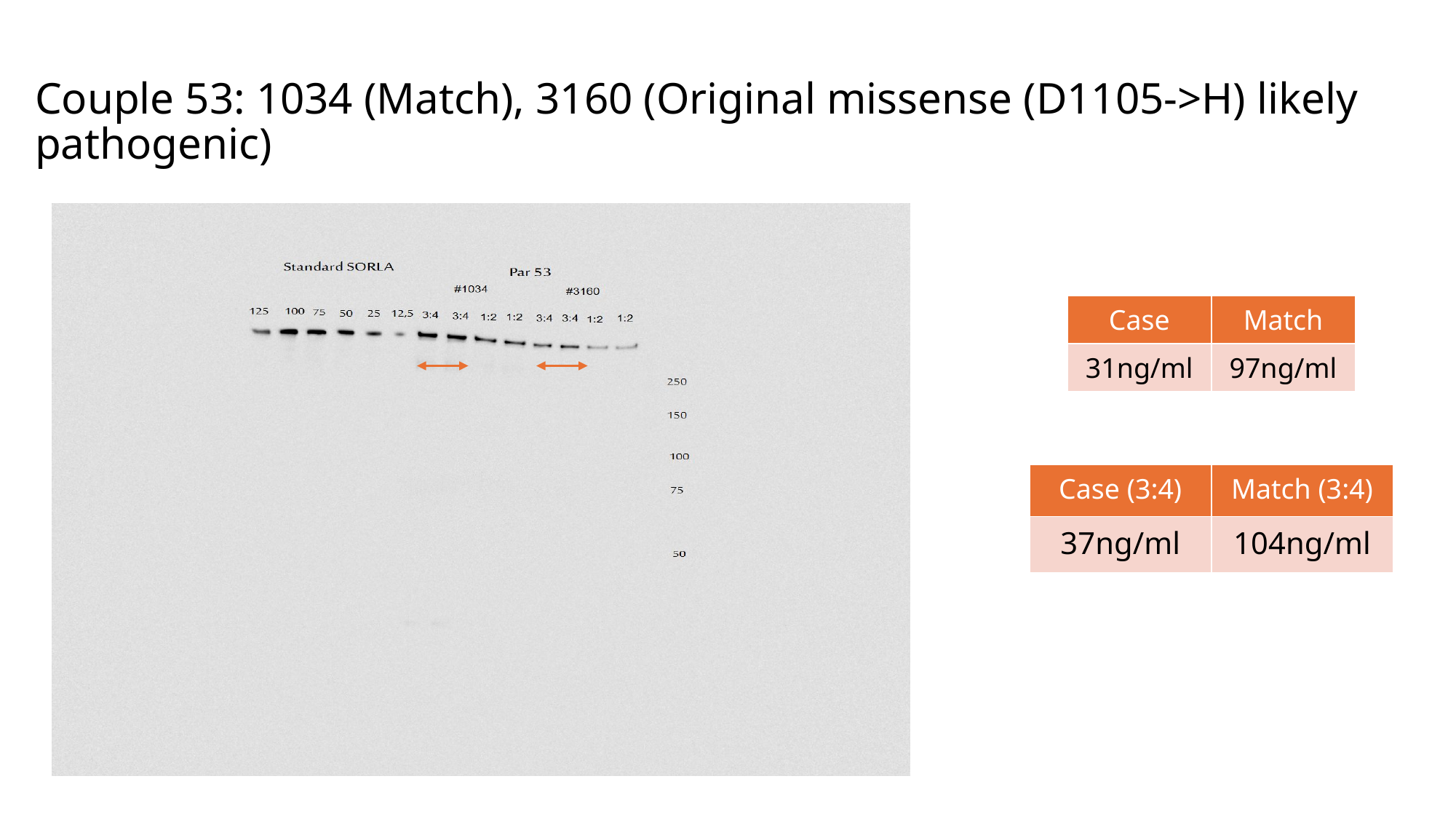

# Couple 53: 1034 (Match), 3160 (Original missense (D1105->H) likely pathogenic)
| Case | Match |
| --- | --- |
| 31ng/ml | 97ng/ml |
| Case (3:4) | Match (3:4) |
| --- | --- |
| 37ng/ml | 104ng/ml |

## Slide 4
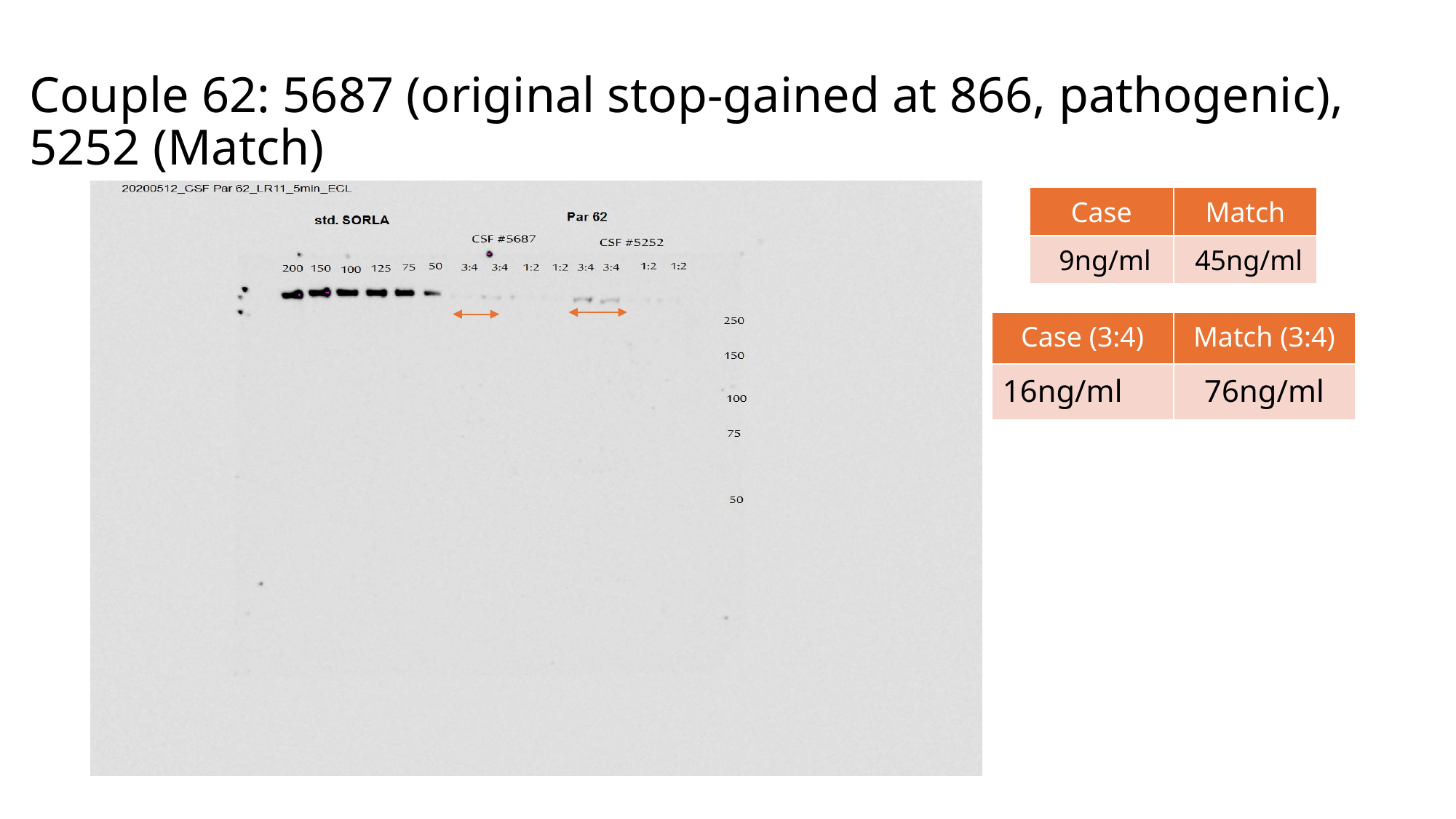

# Couple 62: 5687 (original stop-gained at 866, pathogenic), 5252 (Match)
| Case | Match |
| --- | --- |
| 9ng/ml | 45ng/ml |
| Case (3:4) | Match (3:4) |
| --- | --- |
| 16ng/ml | 76ng/ml |
