# Supplementary material for: Soluble SORL1 in cerebrospinal fluid as a marker for functional impact of rare SORL1 variants
Source: Alzheimers Dement. 2026 Feb 13;22(2):e71042. doi: 10.1002/alz.71042 (PMC12902900; doi:10.1002/alz.71042)
Supplement: Supplementary file 4 — Supporting information [file ALZ-22-e71042-s002.pptx]

## Slide 1
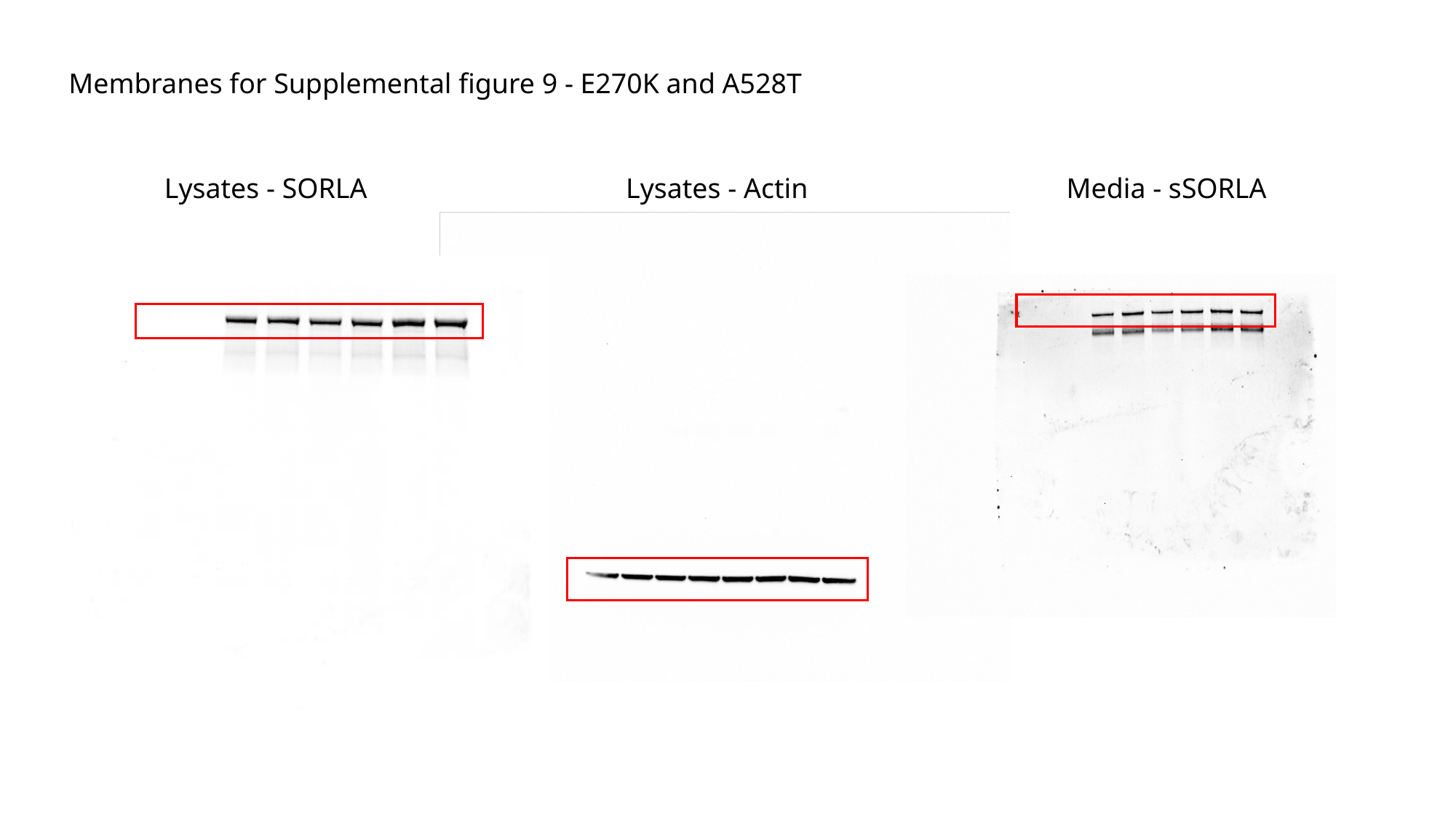

Membranes for Supplemental figure 9 - E270K and A528T
Lysates - SORLA
Lysates - Actin
Media - sSORLA

## Slide 2
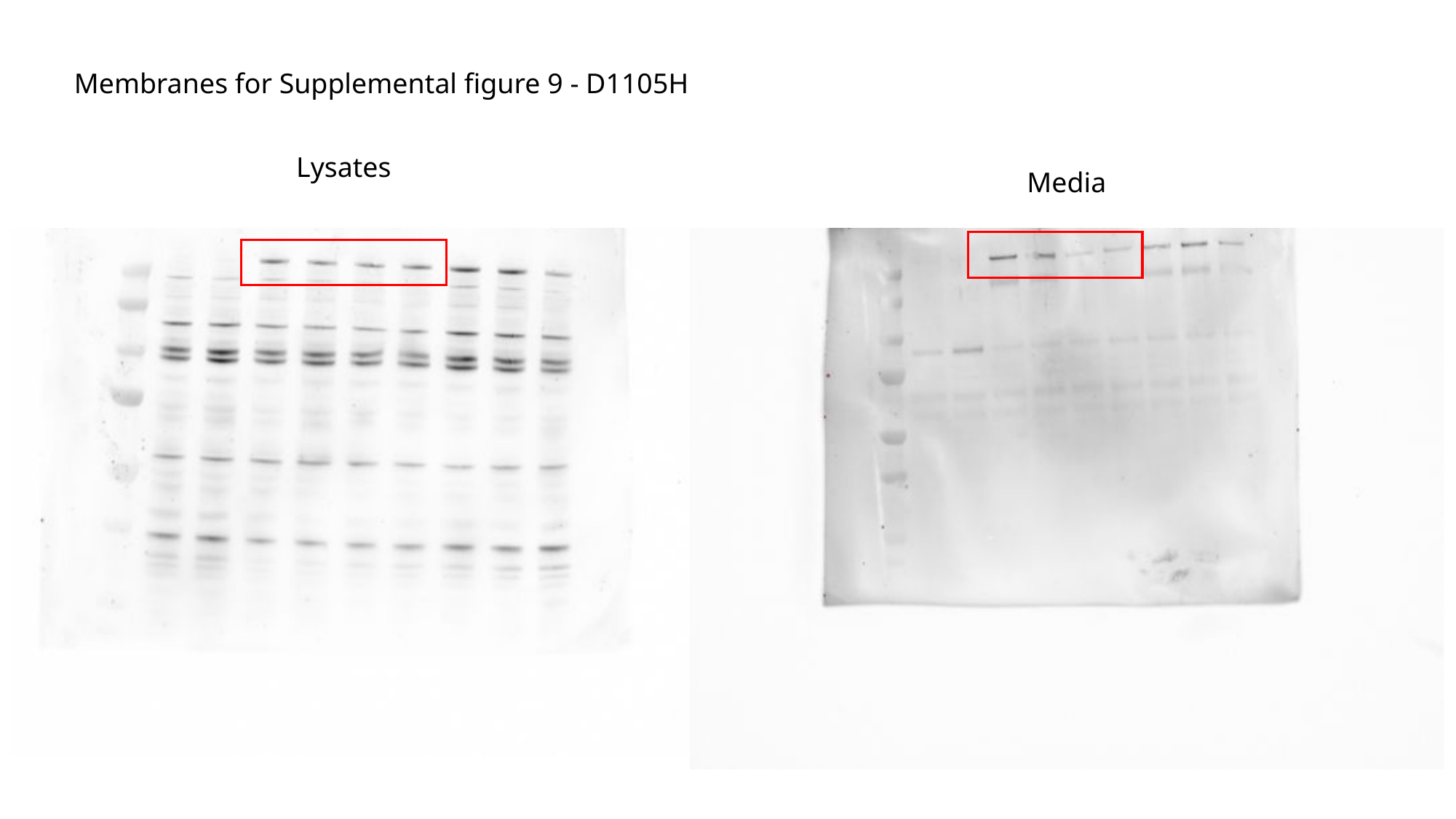

Membranes for Supplemental figure 9 - D1105H
Lysates
Media

## Slide 3
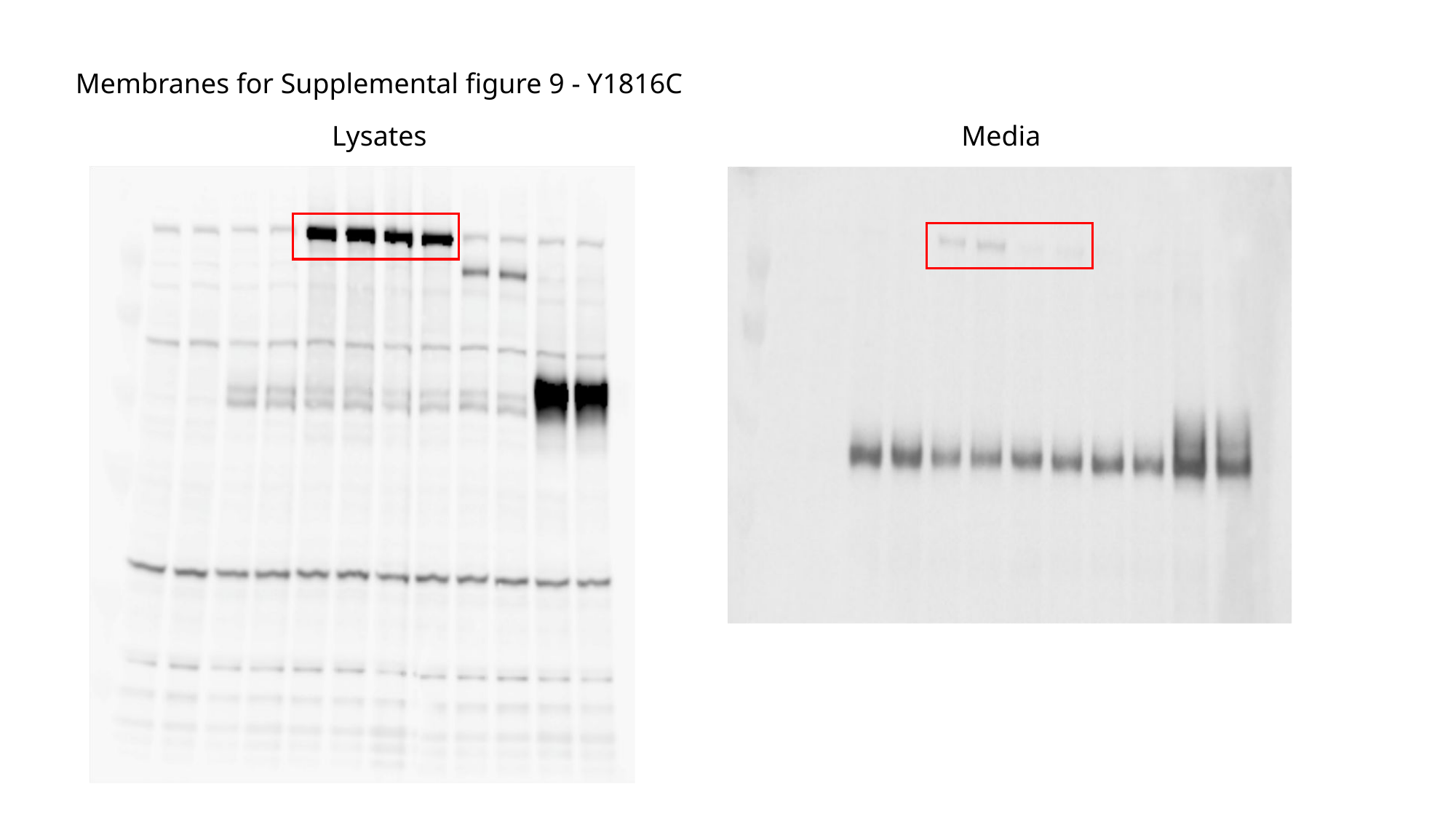

Membranes for Supplemental figure 9 - Y1816C
Lysates
Media

## Slide 4
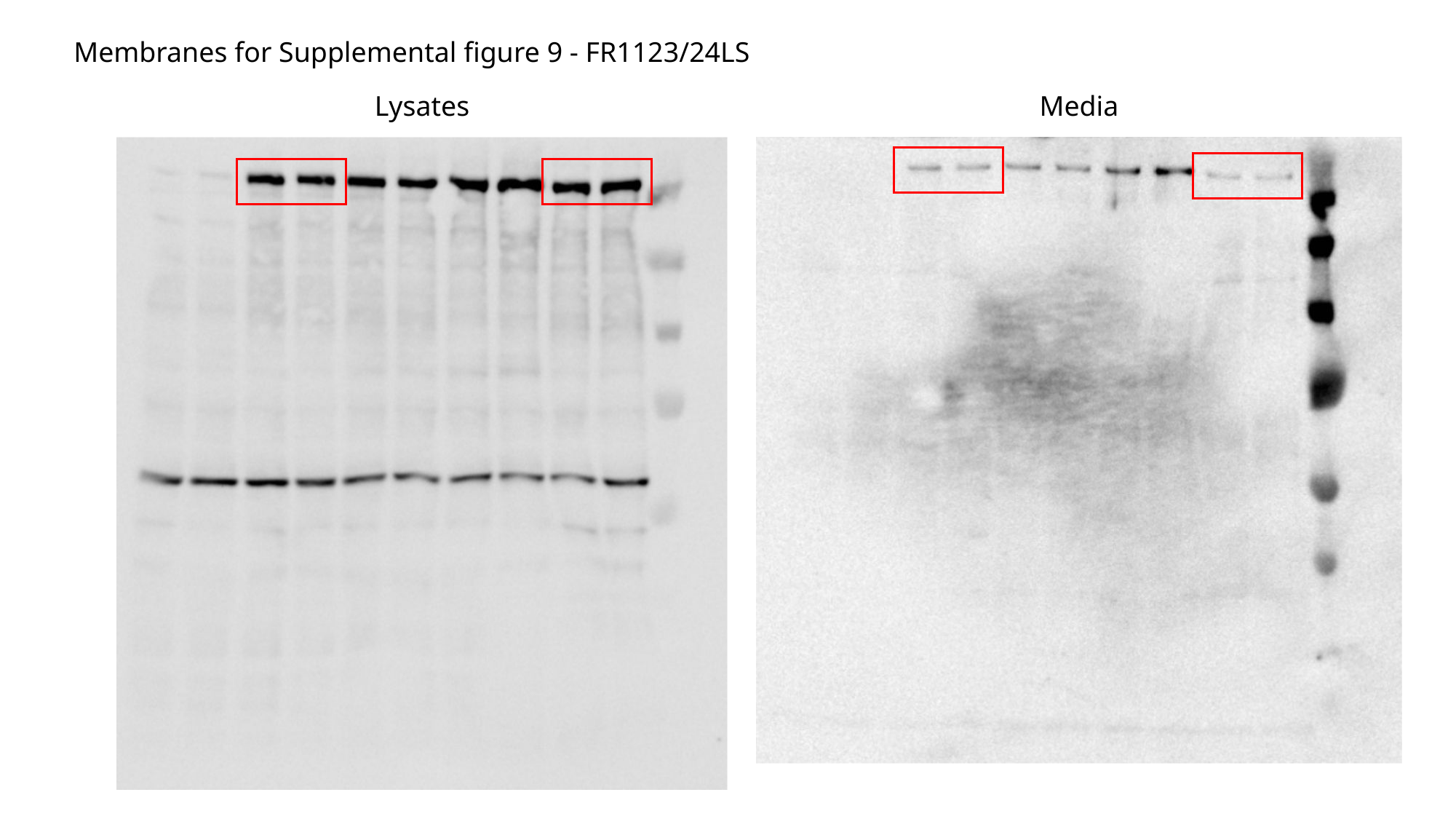

Membranes for Supplemental figure 9 - FR1123/24LS
Lysates
Media
